# Adopting recommendations for implementing patient involvement in cancer research: a funder’s approach

**DOI:** 10.1186/s40900-023-00410-z

**Published:** 2023-03-01

**Authors:** Alexandre B. Costa Alencar, Wendy K. D. Selig, Jan Geissler, Tamás Bereczky, Alba Ubide, David Haerry, Richard Stephens, Valerie Behan

**Affiliations:** 1 RTFCCR, Schaffhausen, Switzerland; 2 Rising Tide Foundation, WSCollaborative, McLean, USA; 3 Patvocates, Hohenbrunn, Germany; 4 Patvocates, Bern, Switzerland; 5 NCRI Consumer Forum, London, UK

**Keywords:** Clinical research, Patient involvement, Philanthropic funding, Grant making, Funding guidelines, Focus areas, Call for proposals, Grant review process, Patient directed trials, Low- and middle-income countries

## Abstract

**Background:**

The role of patients in cancer research is undergoing a significant evolution as all stakeholders seek to enhance the level of direct patient involvement in the design and development of clinical trials. However, there are significant hurdles that patients, patient advocates, laboratory researchers, clinical investigators, and funding institutions must overcome to implement relevant patient involvement in all aspects of biomedical research. By using innovative grant funding models, philanthropic organizations can lead the field in overcoming these challenges. Rising Tide Foundation for Clinical Cancer Research (RTFCCR), a private philanthropy that funds academic research, has developed a novel approach for requiring and supporting partnerships among grantees and patients in designing and conducting research projects. This paper presents a reflective case study of efforts to advance the field of patient involvement in clinical research.

**Methods:**

The decision to focus on patient involvement stems from an expressed focus area established by the RTFCCR board of directors. In conducting this work, RTFCCR partnered with Patvocates, a patient advocacy and engagement network, to create a set of guiding documents and resources aimed at public and private health research funders within various national, international, and therapeutic settings. This effort included a landscape assessment, interviews with experts, and an iterative development process.

**Results:**

To date, RTFCCR has completed and disseminated three guiding documents, one for funders, one for grant applicants, and one for patient advocates. These resources have already generated three major ongoing initiatives at RTFCCR: (1) inclusion of these recommendations in the foundation’s funding guidelines; patient input to prioritization of research focus areas; and in topic selection for calls for proposals; (2) direct involvement of patient experts in the grant review process; and (3) a commitment to support high impact clinical research projects in Low- and Middle-Income Countries. Moreover, the foundation has launched a partnership with the International Cancer Research Partnership, the global alliance of cancer research organizations.

**Conclusion:**

By using its grantmaking function and developing standardized approaches for implementation of patient involvement, RTFCCR is seeking to advance patient-centric cancer clinical research. This approach will continue to develop as it is implemented and shared with partners throughout the world.

**Graphical Abstract:**

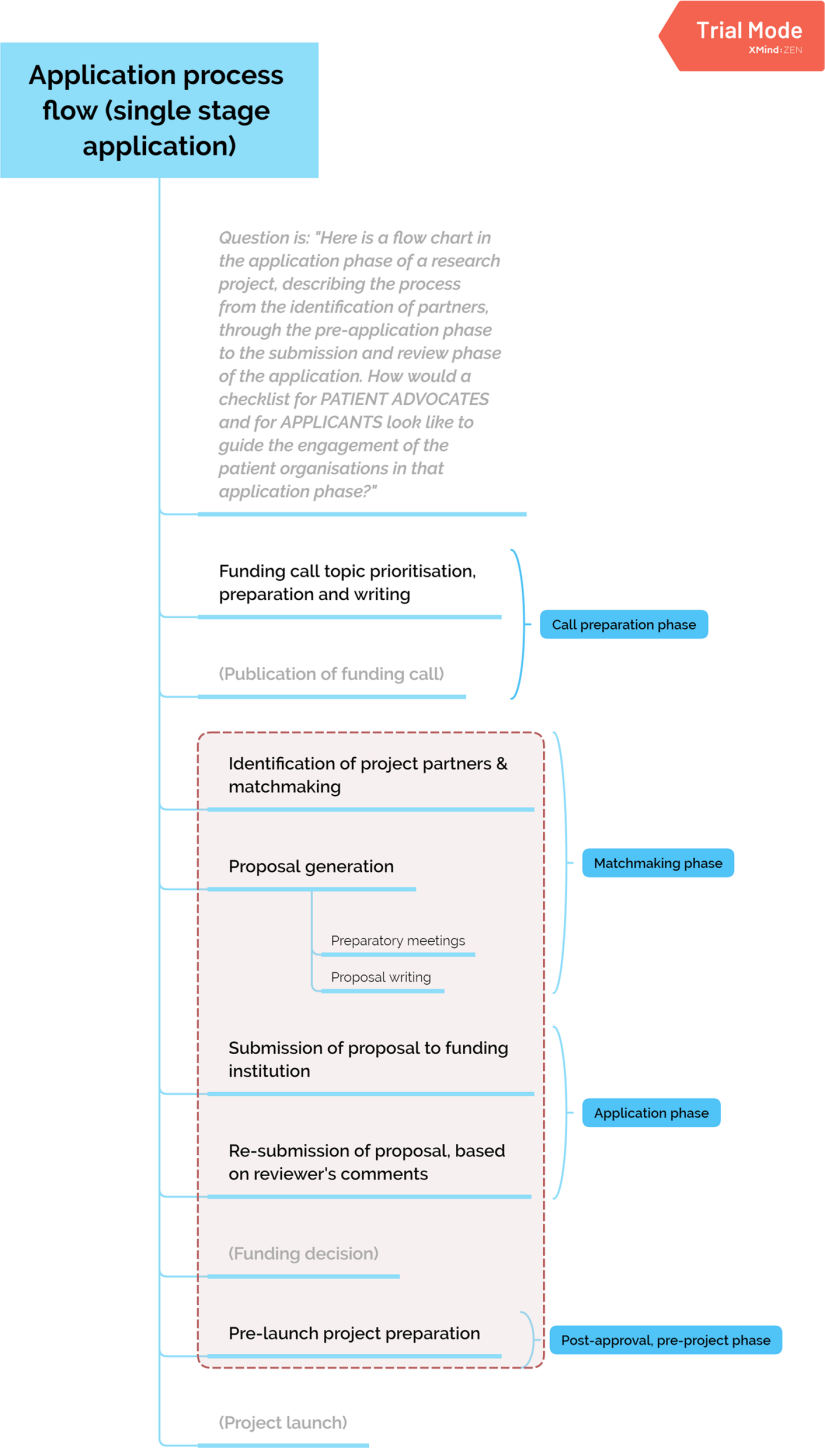

## Plain English summary

The Rising Tide Foundation for Clinical Cancer Research (RTFCCR), a private philanthropy that funds academic research, has developed a novel approach for requiring and supporting partnerships among grantees (scientists) and patients in designing and conducting research projects.

The decision to focus on patient involvement stems from an expressed focus area established by the RTFCCR board of directors. In conducting this work, RTFCCR partnered with Patvocates, a patient advocacy and engagement network. Patvocates conducted a landscape assessment, interviews with experts, and their collective experience as patient advocates. This work generated a set of guiding documents and resources. These resources are to help public and private health research funders to better understand current challenges and support scientists and patients through their funding mechanisms.

Three guiding documents, one for funders, one for grant applicants, and one for patient advocates are now available for download at the RTFCCR website: https://www.risingtide-foundation.org/clinical-cancer-research/patient-engagement#start

Delivering a paradigm change involves not only the introduction of additional requirements and rules, but also enhanced education of patients and investigators. By using its grantmaking function and developing standardized approaches for implementation of patient involvement, RTFCCR is seeking to advance patient-centric cancer clinical research.

Development and implementation of consistent policies and procedures for the integration of the patients’ view in the design and review of research proposals is needed for funders as well as for research institutes, both public and private.

## Background

Involving patients in identifying health priorities and outcomes desired from health interventions is increasingly seen as critically important by the clinical research ecosystem. While the concept of ‘patients as partners’ in health decisions is not new, until recently it has been difficult to implement this approach holistically across the clinical research enterprise and it has been largely and pragmatically focused on decision-making at the point of care. A growing consensus is emerging that patient involvement^1^ is critical to clinical research and should happen as early as possible. A patient-centric culture incorporating early patient involvement in design and execution of clinical research fosters innovation and collaborative attitudes that ultimately lead to identification of the best solutions, including design of outcome measures and clinical study protocols that reflect what matters most to patients [1–4]. Furthermore, patients and their caregivers may have more confidence in research outcomes if other patients have provided input to their development [5].

However, there are significant hurdles that patients, advocates, and clinical investigators must overcome to implement relevant, useful, and effective patient involvement in all aspects of the research process.

For example, since patients and patient advocates are usually not embedded as part of the staff of a research institution, they are generally not provided with the logistical support, training, and tools they might need to contribute effectively to the research process. Support tasks that an institution would need to conduct in implementing patient-centric research studies (e.g., providing training and tools, setting up working group meetings, following-up directly with patients to engage their perspectives, maintaining registries, or establishing a social media presence) are essential to facilitating useful patient involvement in research. Yet these activities are often not covered by philanthropic (or any) grant funding because they are categorized as overhead or tasks that can otherwise be completed on a volunteer basis [6].

The establishment of cultures and processes to ensure routine implementation of patient involvement is critical for integrating the views of patients within medicines development. While this will require significant changes in the way stakeholder organizations traditionally work, there is an opportunity to leverage grant funding to incentivize or mandate these changes. Over time, as these efforts become more routine, sharing best practices, and documenting the tangible benefits of effective patient involvement will be powerful motivators for significant change [5].

**Fig. 1:**
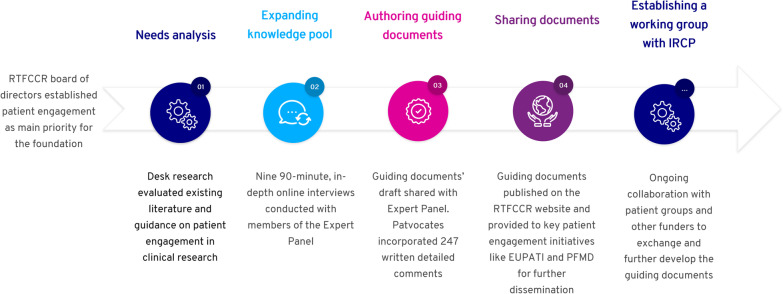
Patient Involvement in Research initiative

By using innovative grant funding models, philanthropic organizations can lead the field in advancing this evolution. Rising Tide Foundation for Clinical Cancer Research (RTFCCR) [7], a private philanthropy that funds academic research, has developed a novel approach for requiring grantees to partner with patients in designing and conducting research projects while supporting them in successfully doing so. This effort stems directly from the vision and mission of RTFCCR [7] and an expressed focus area established by its board of directors^2^ to elevate our patient centricity to a higher aspiration. (Fig. 1).

Examining and understanding recent evidence about the impact and benefits of patient involvement in biomedical research, the RTFCCR Board decided that systematic patient involvement across the organization’s work is not only in line with its original objectives, but also conducive to better and more meaningful outcomes in cancer research. Therefore, the Board commissioned the project on the development of the guidance documents described in this paper on November 02, 2020.

Arising from this work, a set of guiding documents for the three main stakeholders involved in the research process was compiled:

- Patient advocates and patient organizations
- Researchers
- Funders

The guiding documents were developed to close a knowledge gap, providing support for public and private health research funders working in national and international settings. While developed for an oncology area of focus, these model resources provide recommended standard approaches to the implementation of patient involvement from the earliest stages of health-related research that can be customized and applied for use in any disease area. Although RTFCCR’s focus lies in clinical research, the recommendations within this paper are also applicable to advancing patient involvement within basic and translational research, as well as other types of research studies, such as those within the areas of epidemiology or survivorship.

Except for a number of publications and guidance documents on patient and public involvement initiatives in the United Kingdom, current literature and toolkits on patient involvement largely focus on patient interaction with pharmaceutical companies and regulatory institutions on pharmaceutical products.

From approximately 2010 onwards, we have seen a growing body of literature on patient and public involvement in biomedical and health-related research. However, only a small number of non-UK publications on the interaction of the patient community with academic research groups have been published as also confirmed by the description of a systematic research project in Denmark that aimed at developing a guidance for public and patient involvement (PPI) in health-related research [8]. A systematic scoping review of the state of play in PPI in pharmaceutical research found that PPI is the least common in the stages of setting up R&D programs, while PPI in later stages appears to be more widespread [9]. RTFCCR is active in the assessment and funding of research programs in cancer, therefore it is interested in making sure that meaningful PPI already happens in these early stages.

Another scoping review of stakeholder involvement in research priority setting in general [10] found that the involvement of consumers (patients) as a stakeholder group is most widespread in health-related research, however, it also states that only 56% of the research projects set broad stakeholder involvement as a goal. However, most characteristically, it also states that only 1% of the reviewed research projects involved the public (in our case patients, family members, carers etc.) as members of formal governance structures. Another statement in the same review says that “apparently, involving stakeholders in research priority setting can only be ensured if the corresponding funding and support organizations and structures are present”, a finding that this paper also considers important and contains recommendations for.

This paper presents a reflective case study of the efforts undertaken by RTFCCR to develop these resources and advance the field of patient involvement in clinical research.

## Methods

RTFCCR partnered with Patvocates [11], a patient advocacy and engagement network, to identify gaps in current oncology research patient involvement that could be addressed by a grant funder, and build processes to leverage funding frameworks, grant applications, application review, project conduct and dissemination of result to fill those gaps.

Initially, Patvocates conducted desk research to evaluate existing literature and guidance on patient involvement in clinical research, building upon prior literature reviews conducted by Patient-Focused Medicines Development (PFMD) and European Patients’ Academy on Therapeutic innovation (EUPATI), as well as additional publications that were published thereafter.

There has also been limited information [12] about involving patients in research funding bodies, except for the Innovative Medicines Initiative (IMI) Patient engagement Guide and the 600 case studies on Patient Engagement Initiatives published via PFMD Synapse [13, 14]. The analysis of a survey in Australia [15] shows that “consumer involvement may occur only upon request from an external body i.e., research funders”, which also shows that funders (public or private) can play an active role in promoting PPI in research.

To expand on the available resources in this area, RTFCCR and Patvocates created an Expert Panel comprised of individuals with demonstrated expertise in the fields of patient engagement and involvement and medical research to provide input into the process of developing the model approach (Table 1).

**Table 1:** Expert panel members*

Academic researchers	Funders	Patient advocates
Cordula Landgraf (CH) Swiss Clinical Trial Organization Ingrid Klingmann (BE) European Forum for Good Clinical Practice David Gerber (USA) UT Southwestern Medical Center Lidewij Eva Vat (NL) Vrije Universiteit	Michel Goldman (BE) Innovative Medicines Initiative	Mary Lou Smith (USA) Research Advocacy Network Dominique Hamerlijnck (NL) Dutch Lung Foundation Bettina Ryll (SE) Melanoma Patient Network Europe Richard Stephens (UK) National Cancer Research Institute Consumer Forum
**Patvocates experts**		
Jan Geissler Tamás Bereczky, PhD David Haerry		

*The expert panel was composed of renowned patient engagement experts in patient organizations, funding institutions and academic research institutions in the USA and Europe, with all of them having a long track record in patient engagement in related initiatives e.g. EUPATI, PFMD, PARADIGM, WECAN, EFGCP, INVOLVE and Research Advocacy Network. The expert panel was complemented by five decades of experience in patient advocacy and research involvement by the Patvocates team

Nine 60–90-min, in-depth, semi-structured online interviews were conducted by Patvocates and RTFCCR. Interviews comprised 17 questions (Table 2), with different variations for different stakeholder groups. Specific questions were developed by Patvocates and RTFCCR staff based on their experience and evaluation of current literature in five key areas:

- Patient involvement models and resources
- Approaches for identification of and outreach to suitable patient partners
- Patient involvement in development and implementation of calls for proposals
- Assessment criteria for patient involvement in project proposals
- Involvement of patient experts as grant reviewers

**Table 2:** Interview Structure*

Section	Topic explored
Involvement models and involvement guides	Principles of collaboration with patients in research programs
	Models of patient involvement in funded programs
	Funding of patient organizations during application phase (pre-application grant)
	Checklist on involvement of patient organizations and patient advocates during the application phase
Matchmaking between applicants and patient advocates: How to reach out & identify suitable partners	Suggested match-making process between applicant and patient community
	Matchmaking platform
Patient involvement in CALL TOPIC definition, scope and implementation of call for proposals	Process for co-creating request for proposals to ensure patient-centric funding strategy and patient relevance of calls
	Defined measures to avoid conflict of interest of patient expert advisors
Assessment criteria for patient engagement in project proposals	How to rate / evaluate proposed patient participation in projects
	Good practices, metrics and scoring system to assess those criteria
Patient experts as grant reviewers in evaluation process of applications	Handbook for patient advocates to engage in funding reviews
	Guidance for funders on developing pool of patient experts as reviewers
	Training strategy for patient expert reviewers
	Compensation of patient expert reviewers

*Further details on the questions can be found in the appendix

The Patvocates team members also contributed to the content with their expertise in this topic. They summarized and analyzed interview responses through an iterative process. Interview notes were shared with the three researchers at Patvocates to evaluate and summarize the findings through simple manual content analysis that was possible due to the relatively small size of the corpus. Based on these findings, RTFCCR conducted follow-up discussions with colleagues from key funding partner organizations, including the Leukemia & Lymphoma Society (LLS), USA; Melanoma Research Alliance (MRA), USA; and Anti-Cancer Fund (ACF), Belgium. All relevant feedback was synthesized and compiled into the final drafts of the guidance materials, which were then refined and finalized with the assistance of a medical writer.

## Results

### Interview key findings

- There is a need for a paradigm change, involving not only the introduction of additional requirements and rules, but also thorough education of patients and early career researchers.
- There is a need to overcome resistance within academia and among corporate leaders to the notion that patient experts must be treated equally and valued for their contributions in similar ways as other experts.
- More consistent and persevering communication efforts about patient involvement are needed, including through development and implementation of consistent policies and procedures for the integration of patients’ views within the design and review of research proposals.
- While early involvement is important, it is often missing. This type of involvement means including patients in the definition of calls for research proposals and setting research objectives.
- There is a potential benefit in establishing a semi-automated and non-commercial database of patient organizations, patient experts, and lay patients who can be matched to appropriate research projects and initiatives.
- Training is vital and should include resources for mixed method learning approaches such as online activities, hands-on experiences, and the opportunity to work through mock situations.

### RTFCCR guiding documents

The guiding documents were authored based on patient involvement models and methodologies established by leading collaborative initiatives on patient involvement and engagement, e.g., EUPATI, PFMD, PARADIGM (an IMI initiative) and NIHR-INVOLVE, the practical experience of the Patvocates team engaged in collaborative research projects funded e.g., by IMI, Horizon 2020 and other programs, and the input received in the expert interviews. In March 2021, the first drafts of the guiding documents were shared with the patient engagement experts, who provided 247 written detailed comments. These were then incorporated into the documents by Patvocates in May and June 2021. The final drafts were then reviewed by the Patvocates and Rising Tide Foundation team in multiple review cycles before finalization.

In July 2021, the guiding documents for funding institutions and research teams were published on the RTFCCR website and provided to key patient engagement initiatives like EUPATI and PFMD for further dissemination as well as presented at the International Cancer Research Partnership (ICPR) workshop in September 2021. The guiding document for patient organizations was released on the RTFCCR website in December 2021.

Multiple gaps, concerns, and challenges for patient advocates, researchers [16, 17], and funding organizations were identified, including:

Researcher challenges:

- *Feared loss of control* including concern that, through patient engagement, researchers may relinquish part of their control over the research process which they often regard as their “territory.”
- *Need for change* including concern that the research processes applied over years will need to change/adapt to the new situation and require new models of operation.
- Limited resources including concern that budget for involvement of patients in the preparatory research phase is usually not available, and the fear that patient involvement limits available resources for the actual research budget.
- *Deadline pressures* including concern about tight timelines for submission of grant applications in response to calls for proposals.
- *Lack of knowledge* including lack of understanding about how to approach patients, which methodologies to use to extract appropriate feedback for a specific research project, and where exactly patient input would be most beneficial.

Patient challenges:

- Volunteer time and resource constraints including concern that a dedicated budget is needed to cover expenses and widen the pool of patients to avoid over-reliance on a small number of patients.
- *Preparedness barrier* including concern, especially among volunteer groups that may not prioritize involvement in research projects, that they may not have the expertise or time to pursue this work.
- Barriers to access to training including concern that additional resources and funding for training are needed.
- *Need for recognition and acknowledgement* including concern that patients will be acknowledged in publications arising from research that they had helped to shape.

Funder challenges:

- *Changing Organizational Culture*: including concern that organizations with no current involvement may not know where to start, especially when the organization funds basic or non-clinical, research. Training is needed for researchers and grant review panels in best practice in patient engagement.
- *Evaluation and Evidence Development*: including concern about the difficulty of evaluating and measuring success of patient engagement.
- *Access to Diverse Patients*: including concern about engaging a wider pool of patients with lived experience of specific cancer types and experience in strategy and grant reviewing.

The guiding documents addressed these various challenges in the following topic areas:

## Patient engagement for funding institutions

- *Patient engagement in prioritization and generating topics for calls for proposals* Provide recommendations for how funders could engage with patient advocates when compiling calls for proposals, to make sure patient relevance is considered in the text of the call for proposals (CFP), and to define the requirements applicants would need to fulfill in terms of their patient engagement strategy when responding to a specific call for proposals. Underscore that patient engagement should also be ensured in the dissemination of published CFPs in the patient community to raise their interest to collaborate with researchers in applications.
- *Bringing researchers and patient communities together* Provide recommendations for how funders can facilitate researchers to identify relevant patient partners for the application and, should the project be granted, for implementing their project. This includes providing pre-application funding for patient engagement and underscore ways that funders could support patient organizations and advocates with their effort during the application phase before a project is being funded, for example with grant funding specifically earmarked for the time or tasks needed to accomplish these activities.
- *Patient engagement in assessment of applications* Provide recommendations for how funders could engage with patient experts as review panel members when assessing grant applications and making recommendations for funding.

## Patient engagement for applicants

- *Checklist for applicants when planning patient engagement* during the application phase, during the implementation of the project, and beyond the project: This is an activity checklist when planning patient engagement in the application phase, during the implementation of the project, and beyond the project.
- *Examples for potential contributions of patients, caregivers, patient advocates and patient experts* to a research project: The list is adapted from the Drug Information Association (DIA) recommendations on the different roles and functions of patients [18].
- *Organizational models of patient engagement in research projects*, including patient roles helping with coordination, contribution, and provision of advice for a study: This section is designed to help the applicant team and the patient community agree on a meaningful model on patient engagement for a specific research project. It is based on the classification of patient roles and contributions described above. Examples are also added from previous research projects that include relevant patient engagement and input.
- *Identification of patient advocates as research partners* with the best knowledge and insight into the patient population and its subpopulations that will be subject of this research: This section is designed to help the applicant team to identify patient organizations or patient advocates who are the best candidates to achieve the objectives of a patient-relevant clinical research project. The section also describes limitations in terms of resources available to patient organizations and patient partners.
- *Patient engagement plans*, describing patient engagement processes during application and implementation of a research project: This section describes important aspects that must be included, like patient engagement strategies and processes during the project implementation. It describes ways to engage with and identify useful perspectives among the patient community when defining research questions, writing the grant proposal, submitting the grant, revising the proposal, and implementing the project.
- *Preparing the patient community* for its contribution in the post-application, pre-launch phase: This section discusses considerations for involving the patient community in preparing the launch of the project before official funding begins. Given that the funding is generally not available before the official project launches, resourcing from patient partners for preparatory work will likely be limited.

## Recommendations for patient organizations and patient advocates on their involvement in collaborative research projects (Table 3)

**Table 3:** Patient engagement roles

Role	Description	Impact, effort, pros, cons
Project coordinator	Patient organization leads and coordinates the whole project	Impact: very high Effort level: very high ** + **Most influential role, e.g., patient-led research project **–** Highest workload, skills, experience and commitment required Example: European Patients’ Forum in EUPATI, https://www.imi.europa.eu/projects-results/project-factsheets/eupati
Steering committee member	Patient organization / advocate is member of the governing committee of the project—and is funded for the work delivered	Impact: very high Effort level: very high ** + **Patients are part of all relevant strategic decisions High workload, skills, experience and commitment required Not always funded for the work delivered Example: ART CC, HIV cohort collaborations, http://www.bristol.ac.uk/art-cc/
Work package leader	Patient organization / patient advocate coordinates a specific work package in the project	Impact: high Effort level: high ** + **Patients with responsibility to coordinate and deliver defined elements of the project e.g., a work package on patient engagement, needs assessment, external communication ** + **Patients organizations (sometimes) funded for the work delivered **–** High workload, skills, experience and commitment required Example: LeukaNET in the IMI HARMONY Big Data project, https://www.harmony-alliance.eu/patient-cluster, or Myeloma Patients Europe in SISAQOL-IMI, https://event.eortc.org/sisaqol/
Research project member	Patient organization/patient expert is a full member of the research project	Impact: medium Effort, skills, experience level: medium ** + **Full participant of the overall project team ** + **Patient organizations (sometimes) funded for the work delivered **–** Limited influence on decisions, usually only through project meetings of work packages and annual assembly Example: Association Française du Gougerot Sjögren – AFGS in H2020 NECESSITY, https://www.necessity-h2020.eu/patient-involvement/
Patient engagement hub	Patient organization/patient expert is a full research project member, coordinating contribution from other patient organizations outside of the project team, e.g., indication specific	Impact: high Effort, skills, experience level: high + Full participant of the overall project team + Patient organizations funded for the work delivered + /– Does the administration and coordination workload for the wider patient community Example: LeukaNET in the IMI HARMONY Big Data project, https://www.harmony-alliance.eu/patient-cluster, or Myeloma Patients Europe in SISAQOL-IMI, https://event.eortc.org/sisaqol/
Associated project partner	Patient organization has a partnership agreement with the research project	Impact: low Effort, skills, experience level: medium + Patients may prefer as it may take less time + Easier to combine with other activities – Patient organization usually not funded for the contributions and work – Usually not much influence on decisions of the project – Usually no compensation for time, so little time investment possible Example: Patient Advisory Group of four patient organizations in IMI PREFER, coordinated by ECPC, https://www.imi-prefer.eu/stakeholders/patients/
Advisor / advisory board member	Membership of ethics committee, scientific advisory board, project advisory board, data safety monitoring board	Impact: low Effort, skills, experience level: low + Patients’ expertise provided into specific committees, but no participation in active work – Usually no compensation for time, so little time investment possible – Advice only – usually little influence on decisions and no accountability whether advice is actually used and implemented by project Example: Patient Advisory Group of four patient organizations in IMI PREFER, coordinated by ECPC, https://www.imi-prefer.eu/stakeholders/patients/

- *Organizational models and coordinating, contributing, and advising roles in research projects with examples for potential contributions* of the patient community to a research project: This section provides recommendations to patients, caregivers, patient advocates, patient experts, and patient organizations relating to how they can play a governing, partnering, advising, or reacting role in a collaborative research project. Which role works best for the individual project depends on the desired contribution and engagement level.
- Patient engagement in the *definition of research questions and topics of calls for proposals*, and in the promotion of calls for proposals that patient engagement will happen: This section is designed to help the patient community develop and agree on a meaningful model on patient engagement for a specific research project. It is based on the classification of patient roles and contributions described above. Examples are also added from previous research projects which include relevant patient engagement and input.

- *Identifying researchers, collaborative projects, and patient partners for collaborative projects*, and how they may find each other during the application phase: This section recommends how funders can facilitate the process by which researchers identify relevant patient partners for the application and implementing the project once funding is awarded.
- *Involvement of the patient community during the application phase* before a project has been funded, including models for funding patient contributions during that phase (e.g., with grants covering time or tasks): This section describes aspects of the pre-submission phase in which the collaborating partners compile a thorough and complete proposal for submission to the funding institution and make key decisions on the research question, the objectives and intended outcomes, the overall project structure, its governance, and tasks and responsibilities of all involved partners.
- *Involvement as patient reviewers* Potential assessment questions are proposed to help score applications for the level and quality of their proposed patient engagement. These questions should be listed in the application guide to help applicants in developing their patient engagement plan for their grant application.

## Impact

At the time of writing, the guiding documents described above have already catalyzed three major ongoing initiatives at RTFCCR:

## Patient engagement in research embedded in our funding strategy (Fig. 2)

**Fig. 2:**
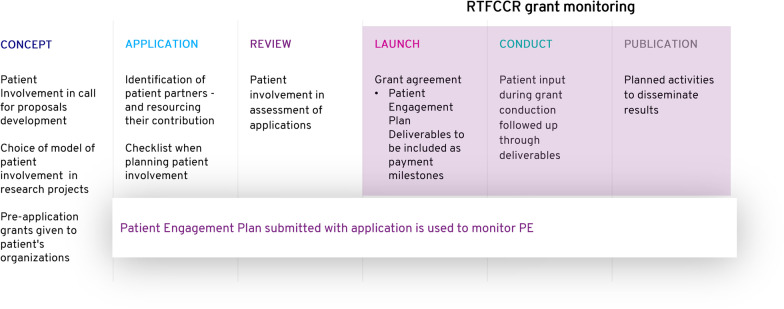
Changes made to the RTFCC funding based on the recommendations developed in this project

The essence of these recommendations has been included in the foundation’s funding guidelines to ensure its grant making process is inclusive and supportive of patients. A Patient Engagement Plan is also now required as part of every grant application package and is used to define payment milestones in every grant agreement.

In a proactive approach, RTFCCR now includes patients in the process for providing input to prioritization of research focus areas and topic selection for calls for proposals. Furthermore, the foundation is including patient engagement not only in its open application calls but also in all collaborations, including joint calls for proposals with ACF, MRA and SCR (Swiss Cancer Research—Krebsforschung Schweiz) for patient-directed clinical trials.

Lastly, a new type of grant has been established, the Pre-Application Grant. These grants, ranging from 1000 to 4000 Euros, are designed to close the funding gap for patient experts, allowing them to provide input into the development of a grant application/protocol. The goal of this new grant mechanism is to support patient organizations with needed funding for this early phase of engagement. The budget should be planned to cover travel costs to preparatory meetings and the work time invested by staff or patient experts. This work should be carried out as a preparation step for eventual submission of a clinical research grant application to RTFCCR.

## Direct engagement of patient experts in the grant review process

RTFCCR has established a Patient Partner Sounding Board. This is a selected group of nine patient experts from Europe and US who share their community insights and patient advocacy expertise with the foundation, providing input for multiple research and advocacy initiatives funded by RTFCCR. This group is also contributing to ongoing grant review and strategy discussions.

## Commitment to support high impact clinical research projects in low-and-middle-income countries (LMIC)

In developing this new area of focus for the foundation, a landscape analysis of current patient-centric research funding needs will be undertaken to support eventual partnership with local foundations in launching a geography-based call for proposals to support patient-centric clinical cancer research adapted to unique regional needs in these countries.

The impact of patient input gathered through the new guidances and processes will be collected in the review of completed research projects which were funded under the new patient engagement scheme.

## Discussion

As a general need, it was highlighted that while there is broad multistakeholder consensus on patient involvement methodologies, frameworks and tools developed by EUPATI [19, 20] and PFMD [21], targeted toward industry-led research, there was a lack of systematic implementation of patient engagement models and involvement guides in funding institutions and academic research. Some suggestions have been raised as to who should develop this topic, (i.e., EUPATI, the Swiss PPI Hub, FasterCures). These are seen as hubs for knowledge and experience necessary to bring this vision of patient-centric research to life.

Regarding geographic differences, there are some non-systematic patient training initiatives underway at research institutions, funders, and patient organizations. In the US for example, NCI SPORE and the National Clinical Trials Network (NCTN) are often mentioned as benchmarks and sources of resources for these efforts, while in Europe, the European Patients Academy (EUPATI) has developed a portfolio of online trainings for patient advocates, academia, and industry as well as the EUPATI Toolbox [22] as an educational resource in 13 languages.

The RTFCCR efforts have yielded a strong consensus among stakeholders that there is a need to create a better process for identifying and involving patients who are willing and able to engage in a patient-centric research process. This type of process has been described as a “matchmaking service” potentially managed through a semi-automated tool that could match individual patients with research study teams in a flexible and creative way. At this point, the details of how such a service should be set up, who should run it, or how it will be sustainably funded have not been fully developed.

As a concept, this type of platform would best be undertaken by multiple stakeholders, would be permitted to function independently, and could be controlled by patient communities. The need for such a platform exists both on a global scale and within individual countries or geographies.

Delivering a paradigm change involves not only the introduction of additional requirements and rules, but also enhanced education of patients and investigators.

Development and implementation of consistent policies and procedures for the integration of the patients’ view in the design and review of research proposals is needed for funders as well as for research institutes, both public and private.

The guiding documents discussed here represent a starting point for funders to explore gaps and opportunities for sparking needed changes within their funding culture.

The foundation’s first grant reviews including the principles described here occurred in late 2021. This engagement of the foundation’s team with patient experts has been very informative and effective, addressing challenges not previously identified, allowing a structured assessment of the patient engagement proposed by researchers, and eventually improving the quality of the research being funded.

### Advancing the field of patient engagement and involvement in clinical research

As a next step from this work, the foundation has launched a partnership with the International Cancer Research Partnership (ICRP) [23]. ICRP is a unique alliance of cancer research organizations from Australia, Canada, France, Japan, the Netherlands, United Kingdom, and the United States. The Partners share funding information to enhance global collaboration and strategic coordination of research between individual researchers and organizations.

Through the establishment of a working group, the ICRP members will collaborate with patient groups and other funders to exchange and further develop the guiding documents. The groups’ larger ambition is to influence other funders to embrace this approach to medical research.

Three major topics will be addressed by this group beginning in 2022: (1) Sharing of best practices on patient advocate training, (2) Implementation of patient engagement by group members in different settings (academic, industry, private funders) and sharing of best practice (3) Audit of existing possibilities that can match patient organizations, patient experts and lay patients to research projects and initiatives.

The results of this work will be shared openly on ICRP and RTFCCR’s websites.

## Conclusion

There is much to be done to evolve current approaches to health-related research and make every aspect of those efforts as patient-centric as possible. Engaging a range of research funders in undertaking changes to their funding processes based on the RTFCCR model and recommendations will play a key role in ensuring that our efforts can be scalable with the goal of making patient involvement the new norm for the conduct of global health-related research.

## Data Availability

Not applicable.

## Appendix

See Table

**Table 4:** Interview questions asked to patient advocates, researchers, and funders

Questions	Group asked
Have you come across any systematic guidance for collaboration with patients in research programs that you have been involved in? If yes, please let us know the source	Patient advocates Researchers Funders
The usual roles for patient involvement in research projects are Project coordinator Steering committee member Consortium member Hub model Work package lead Associated partner Advisor/advisory board member Is any role missing? Can you say something about the strengths, weaknesses or each of the models? And how would they be made most effective?	Patient advocates Researchers Funders
Patient organizations often fail to resource a lengthy application phase and pre-application meetings and document revisions. What would a fair process look like to cover those pre-application efforts, and what should be funded and what should not be funded?	Patient advocates Researchers Funders
Here is a flow chart in the application phase of a research project, describing the process from the identification of partners, through the pre-application phase to the submission and review phase of the application (to patient advocates) What would a checklist for PATIENT ADVOCATES look like to guide the involvement of your patient organization in that application phase? (to researchers) What would a checklist for GRANT APPLICANTS look like to guide their involvement of patient organizations during the whole application phase? (to funders) What would a checklist for PATIENT ADVOCATES and for APPLICANTS look like to guide the involvement of the patient organizations in that application phase?	Patient advocates Researchers Funders
(to patient advocates) What would help you to find those that apply for grants in your specific indication area and identify calls that are relevant for your patient organization? (to funders) What could the process of identifying the right patient organization for a grant application/ project look like?	Patient advocates Funders
HOW should a tool or platform support to bring applicant consortia and patient organizations or patient experts together? Are you aware of any existing tools? Should each funder and patient community build one, or would you use a matchmaking tool / pool of patient experts of a third party, e.g., EUPATI, PFMD etc.?	Patient advocates Researchers Funders
What mechanisms would support patient involvement happening in the phase when research topics and calls are defined? Have you seen things that worked and that did not work?	Patient advocates Researchers Funders
(to patient advocates and researchers) Can you think of any Conflict of Interest in patient involvement in funding programs that would be different to Conflict of Interest criteria for academic experts and institutions? What could be done to avoid those in a funding program? (to funders) Can you think of any Conflict of Interest in patient involvement in funding programs that would be different to Conflict of Interest criteria for academic experts and institutions? What could be done to avoid those in a funding program?	Patient advocates Researchers Funders
What is missing in the following questions / assessment criteria that may be applicable to call texts and grant reviews? Would you have other questions that are relevant? How will applicants assess patients’ needs, goals, concerns, or preferences? In which role and function will applicants involve patients in the proposed project? Is patient involvement adequately resourced? How relevant will the project outcomes and value be for patients? How will patients’ acceptance and relevance of the outcomes be evaluated?	Patient advocates Researchers Funders
Are you aware of good practices for measuring and assessing PE in proposals? Have you seen meaningful scoring systems or metrics?	Patient advocates Researchers Funders
If we were to create a handbook for patient advocates on the involvement in the REVIEW of funding proposals to help identify the most promising grant applications, what should be in such a handbook?	Patient advocates Researchers Funders
If a funder would build a pool of patient experts that can be tapped for grant reviews panels, what should that look like?	Patient advocates Researchers Funders
If we would provide training to patient advocates to improve their knowledge and skills to provide meaningful contributions to grant review panels, what would a curriculum need to cover? How much training should there be, and in which format?	Patient advocates Researchers Funders
What would be your advice on the compensation of patient expert reviewers?	Patient advocates Researchers Funders

4

## Abbreviations

RTFCCR: Rising Tide Foundation for Clinical Cancer Research
RFP: Request for proposals
LMIC: Low- and middle-income countries
ICRP: International Cancer Research Partnership
PFMD: Patient-Focused Medicines Development
EUPATI: European Patients’ Academy on Therapeutic Innovation
NIHR: National Institute for Health Research
IMI: Innovative Medicines Initiative
LLS: Leukemia & Lymphoma Society
MRA: Melanoma Research Alliance
ACF: Anti-Cancer Fund
PARADIGM: Patients active in research and dialogues for an improved generation of medicines
NIHR-INVOLVE: Promotes and supports patient and public involvement (PPI) in NIHR-funded research
Horizon 2020: Financial instrument implementing the Innovation Union, a Europe 2020 flagship initiative aimed at securing Europe’s global competitiveness
CFP: Call for proposals
DIA: Drug Information Association
ART CC: Antiretroviral Therapy Cohort Collaboration
HARMONY: Healthcare alliance for resourceful medicines offensive against neoplasms in hematology
NECESSITY: New clinical endpoints in primary Sjögren’s syndrome: an interventional trial based on 21stratifying patients
SISAQOL-IMI: Setting international standards in analysing patient-reported outcomes and quality of life endpoints
ECPC: European Cancer Patients Coalition
PREFER: Patient preferences in benefit risk assessments during the drug life cycle
SCR: Swiss Cancer Research—Krebsforschung Schweiz
NCI SPORE: Specialized Programs of Research Excellence
NCTN: National Clinical Trials Network

## Footnotes

1 Patient involvement means the active involvement of patients, caregivers and patient organizations based on co-design principles that allow a better formulation of patient-relevant research questions and, more effective and relevant data generation.

2 "The RTFCCR Board is composed of six experts from the fields of science, economics, patient advocacy and law. For more information see: https://www.risingtide-foundation.org/clinical-cancer-research/board-members-rtfccr
